# Artificial Intelligence Modeling-Based Optimization of an Industrial-Scale Steam Turbine for Moving toward Net-Zero in the Energy Sector

**DOI:** 10.1021/acsomega.3c01227

**Published:** 2023-06-02

**Authors:** Waqar Muhammad Ashraf, Ghulam Moeen Uddin, Rasikh Tariq, Afaq Ahmed, Muhammad Farhan, Muhammad Aarif Nazeer, Rauf Ul Hassan, Ahmad Naeem, Hanan Jamil, Jaroslaw Krzywanski, Marcin Sosnowski, Vivek Dua

**Affiliations:** † The Sargent Centre for Process Systems Engineering, Department of Chemical Engineering, University College London, Torrington Place, London WC1E 7JE, U.K.; ‡ Department of Mechanical Engineering, University of Engineering & Technology, Lahore, Punjab 54890, Pakistan; § Facultad de Ingeniería, Universidad Autónoma de Yucatán, Av. Industrias No Contaminantes por Anillo Periférico Norte, Apdo. Postal 150, Cordemex, Mérida, 97203 Yucatán, Mexico; ∥ Department of Automotive Engineering Technology, Punjab Tianjin University of Technology, Lahore 54000, Pakistan; ⊥ Faculty of Science and Technology, Jan Dlugosz University in Czestochowa, 13/15 Armii Krajowej Av., 42-200 Czestochowa, Poland; ⊗ Tecnológico Nacional de México/IT de Mérida, Departamento de Sistemas y Computación, Mérida, Mexico

## Abstract

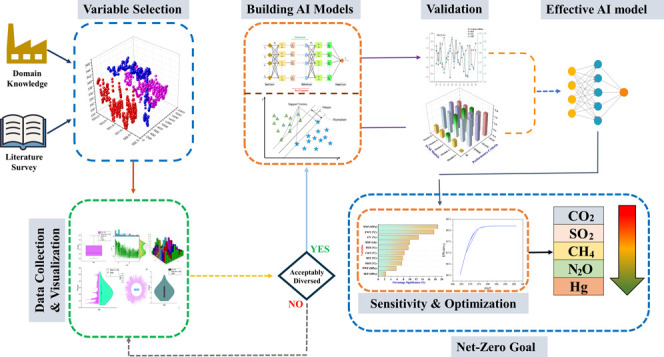

Augmentation of energy efficiency in the power generation systems can aid in decarbonizing the energy sector, which is also recognized by the International Energy Agency (IEA) as a solution to attain net-zero from the energy sector. With this reference, this article presents a framework incorporating artificial intelligence (AI) for improving the isentropic efficiency of a high-pressure (HP) steam turbine installed at a supercritical power plant. The data of the operating parameters taken from a supercritical 660 MW coal-fired power plant is well-distributed in the input and output spaces of the operating parameters. Based on hyperparameter tuning, two advanced AI modeling algorithms, i.e., artificial neural network (ANN) and support vector machine (SVM), are trained and, subsequently, validated. ANN, as turned out to be a better-performing model, is utilized to conduct the Monte Carlo technique-based sensitivity analysis toward the high-pressure (HP) turbine efficiency. Subsequently, the ANN model is deployed for evaluating the impact of individual or combination of operating parameters on the HP turbine efficiency under three real-power generation capacities of the power plant. The parametric study and nonlinear programming-based optimization techniques are applied to optimize the HP turbine efficiency. It is estimated that the HP turbine efficiency can be improved by 1.43, 5.09, and 3.40% as compared to that of the average values of input parameters for half-load, mid-load, and full-load power generation modes, respectively. The annual reduction in CO_2_ measuring 58.3, 123.5, and 70.8 kilo ton/year (kt/y) corresponds to half-load, mid-load, and full load, respectively, and noticeable mitigation of SO_2_, CH_4_, N_2_O, and Hg emissions is estimated for the three power generation modes of the power plant. The AI-based modeling and optimization analysis is conducted to enhance the operation excellence of the industrial-scale steam turbine that promotes higher-energy efficiency and contributes to the net-zero target from the energy sector.

## 1 Introduction

Electric power consumption plays a critical role in the industrialization of any country.^1^ In 2021, global electric power consumption has increased by 4.5% despite the COVID-19 crisis.^2^ According to the key world energy statistics report published by the International Energy Agency (IEA), 63.1% of the total energy supply was accounted by fossil fuels and coal contributed nearly 36.7% to the worldwide power generation in 2019.^3^ The energy sector is one amongst the major drivers of CO_2_ emissions in the atmosphere that would remain significant till 2050 for the underdeveloped countries as underlined in the recent IEA report on net-zero by 2050.^4^ Till 2050, the global electricity demand will be increased by 80%, out of which more than 85% share will be served for underdeveloped countries.^4^ Despite the growth in renewable energy-based power generation systems, coal would share a significant contribution in meeting the energy demand of underdeveloped countries till 2050. Further details on the total electrical power production and total CO_2_ emission trend based on different regions of the world (refer to Figure S1) are provided in the Supporting Information. The sustained dependence on coal is due to the factors like coal being a low-cost fuel, its well-established energy conversion technologies, the relatively quicker installation of coal-fired power plants, and socioeconomic and political challenges in emerging economies.^4^

The IEA report on net-zero emissions from the energy sector has suggested potential technologies and solutions for decarbonizing the energy sector. However, it is also highlighted that the technology development and installation would be realized in the future depending upon the global commitments and awareness among the communities toward climate change.^4^ Furthermore, the report also underlines the need to operate the existing fossil fuel-based energy assets more efficiently since the higher-energy efficiency offers the same energy service with lesser fuel consumption and reduction in the emission load.^4^ The energy efficiency of the coal-based power systems is particularly important since it accounts for a 40% reduction in energy-related hazardous gas emissions according to IEA’s sustainable development scenario.^4^ It is worth noticing that coal-based energy conversion technologies have become mature enough in the past decades of technological advancement. Therefore, IEA highlights the need to develop better operational and maintenance practices, which require no capital investment and, at the same time, can better run the existing fossil-based power complexes.

The development of an efficient operational strategy for the optimal control of processes and energy systems synchronized with the power generation operation of a large-scale coal power plant is a challenging task because of the involvement of a large number of operating parameters. Such plants typically operate on coal-fired supercritical pressure steam generators integrated with multistage steam turbines and electrical power generators. This complex industrial process and its critical components have been traditionally studied and analyzed using classical textbook analytical models, which are known to have accuracy limitations. Despite the fact that modern coal-fired power plants are equipped with very sophisticated sensory information systems recording operational control and performance factors, the data they record has been severely underutilized. At best, the managements of these power plants graphically or statistically analyze the critical to business performance factors and make conservative decisions. The lack of advanced data visualization, modeling, and mining skills of the traditionally qualified mechanical and electrical engineers is the fundamental impediment to real utilization of this valuable data. Assisted with modern artificial intelligence (AI) techniques, the data of these sensory information systems can be used to develop functional cause and effect models at component, system, and strategic levels that are superior as compared to traditional analytical models in the sense that they are built on the actual information the system is spilling in real time and they can be used to make engineering and management decisions.^4^ Such AI models and their information mining potential have a real potential of serving as the heart and soul of the next-generation data-driven operational management of such industrial systems in the true spirit of industry 4.0 and contributing to net-zero targets from the industrial sector.

The steam turbine is the heart of a power generation process, and its operation is critically controlled for smooth power production, energy economy, and efficiency of the power plants.^5,6^ The operation of multiple electromechanical devices is synchronized with the turbine system in a complicated pattern, and thus generating practical operating solutions for such a nonlinear and hyperdimensional system becomes quite challenging. The conventional mathematical equations/models incorporating such a large number of operating parameters for complex and integrated energy devices are difficult to develop. The constraints of practical power production operation, inherent complexity, and degradation in the system cannot be effectively introduced in these models, thereby limiting their applicability. Furthermore, carrying out the simulation and optimization analysis for large industrial systems by the first-principle model can also be computationally prohibitive. The complex function space built on hyperdimensional input parameters is not accurately approximated by the conventional regression-based techniques, thereby limiting their application to model the operation of large-scale industrial systems.

The AI models have been around for the last two decades now, and we have seen them providing solutions in information technology,^7^ health care,^8^ image, and speech recognition applications.^9−11^ However, the researcher has missed the opportunity of utilizing this remarkable tool with its big-check customer, i.e., the conventional large-scale industrial complexes like power generation systems, chemical process industry, oil and gas refineries, etc. AI can be very effectively utilized to cater to the most critical need of such industries, i.e., exploiting the opportunity for cost savings, performance excellence, environmental emission reduction out of the big operational data these industries are generating. The AI research community is falling short on two accounts: (a) most of the research on the application of AI on conventional engineering systems is being published on data generated on lab-scale pilot project based on carefully designed experiments with a guarantee to show interesting scientific findings^12−14^ and (b) most of the research in this area stops at the development of an effective AI-based process model.^15,16^ Unfortunately, both endeavors are of limited value to industrial executes and engineers of big-check customers. However, if we can demonstrate the potential of AI-based methodologies to save time and money in industrial operations at component, system, and strategic levels and top it up with the quantified reduction in environmental emissions, these industries could adopt such methodologies in their industrial operations.

In the last two decades, AI-based data modeling tools have presented a remarkable performance in developing engineering solutions and optimization strategies for large-scale industrial systems overcoming the limitations of mathematical modeling tools.^17−24^ Our research group has also reported performance enhancement solutions developed on the component level, system level, and strategic level of a 660 MW coal power plant using advanced AI modeling tools and statistical techniques.^17,25,26^ AI-based modeling and simulation algorithms can provide accurate results mined out of the high-dimensional and nonlinear interacting features of engineering systems, which can be reliably implemented in the running operation of energy systems.^17^ However, asymmetric and high-dimensional space of the data, development of efficient AI models and their validation, domain knowledge-backed experimental designs, and operating strategies are the challenges to be addressed carefully to exploit the true potential of data and AI algorithms. Since the interpretation of the AI models is a challenging task, the hybrid modeling framework including the physics-based model describing the system is constructed and the AI-based model is developed on the simulated data of the system for conducting the sensitivity and optimization analysis.^27^ In some scenarios, the AI model is integrated within the analytical framework of hybrid modeling for the property prediction, thereby reducing the computational burden and the AI model can also be utilized for making digital twin applications.^28^

The new generation of AI has witnessed its widespread utilization for carrying out modeling and optimization analyses on various scientific and engineering domains of applications.^29−34^ Mrzljak et al.^35^ performed the exergy analysis on a steam turbine of a nuclear power plant for four different operating conditions by using optimization algorithms—simple algorithm, genetic algorithm, and improved genetic simplex algorithm. The maximum exergy efficiency of 85.92% was obtained by an improved genetic simplex algorithm. Kosowski^36^ proposed a general efficient system for designing turbine cascades and stages. The design approach was based on evolutionary algorithms and shown to be efficient and computationally inexpensive compared with computational fluid dynamics calculations. In another study, Kosowski^37^ applied ANN for estimating the spatial distribution of flow properties like enthalpy, pressure loss, velocity, etc., in the steam turbine cascades. However, the parametric sensitivity of the performance parameters of the turbine system was missing. Zhou et al.^38^ deployed an extreme learning model to monitor the performance degradation of the steam turbine regenerative system for ensuring the safety and economy of the coal-fired power plant operation. In another study, the mode of steam distribution for power generation under different scenarios was optimized based on governing valve characteristic modeling.^39^ Zhu et al.^40^ constructed a mixed-integer nonlinear programming model to optimize the operation of various power generation capacity steam turbine networks for the petrochemical complex. In another study, a data-driven robust optimization analysis was performed to optimize the steam power system of a chemical plant under multiscenario demand uncertainty.^41^

Shuvo et al.^42^ used machine learning modeling tools to predict the electric power production of a combined cycle power plant. The critical operating parameters taken from the energy devices like boiler, turbine, etc., were deployed for developing regression-based machine learning models. Zeqiu et al.^43^ developed a hybrid ANN model to determine the optimal operating conditions for the steam turbine installed at a chemical plant. The optimized solutions brought a 1.4% reduction in the cost of steam production without any investment. Guo et al.^44^ trained the ANN model with backpropagation for modeling the main steam temperature of a steam turbine since it has a direct relation with the isentropic efficiency of the high-pressure turbine. The model presented good generalization and approximation performance in simulating the temperature as modeled on the power plant’s nonlinear and hyperdimensional causal parameters. Dettori et al.^45^ developed an ANN model for predicting the output power incorporating the turbine features that could not be monitored directly, like the quality of steam at the exit of the turbine. Fakir et al.^46^ used deep learning techniques like long short-term memory, convolutional neural network and hybrid long short-term memory, convolutional neural network to predict the electrical power output from an industrial steam turbine. The variance score and root-mean-square error of the best-performing model were 98.29% and 0.12 MW, respectively. Tveit^47^ modeled the steam turbine operation of a combined heat and power plant by mixed-integer nonlinear programming. Nguyen et al.^48^ presented a multistep ahead prediction framework built on a long short-term memory network. The operating conditions of the steam generator of nuclear power plants were predicted based on the values of input parameters.

The AI-based studies published in the literature generally report the modeling performance of the algorithms and, in some cases, the optimization results for the lab-scale, pilot-scale, and model-simulated studies.^14,49−53^ The data sets for such studies follow the typical experimental designs, and the performance enhancement of the investigated system is guaranteed with the considered design space. However, the industrial data-driven AI model development, finding the improvement in the already-designed control space with respect to the operational constraints and subsequent contribution to the net-zero goal, is a challenging task that has not been reported and is of particular importance as well as a research gap to demonstrate the potential of AI for the performance enhancement of industrial systems to the industrial community. Furthermore, deploying the AI-based modeling algorithms for having an insight into the state of the operation of the industrial-scale steam turbine is lacking in the reported literature studies that may enhance the understanding of its working. The sensitivity of the operating parameters toward the turbine efficiency is important because it can help maintain the energy efficiency of the turbine within the controlled limits based on optimal set values of significant parameters. However, the sensitivity of the operating parameters on the industrial-scale steam turbine is not reported in the literature studies. Furthermore, the analysis incorporating the AI model integrated into the rigorous optimization environment for maximizing the HP turbine efficiency is lacking in the literature. The AI model-driven optimal values of the operating parameters corresponding to the maximum possible efficiency of the complex multivariate operation of the steam turbine should be estimated that is of particular interest to the industrial community with respect to the technoeconomic and environmental benefits. Moreover, a comprehensive analysis on investigating the higher-energy efficiency and reduced emission load to the environment in response to the optimum operation of the steam turbine synchronized with the power generation systems of the power plant should also be conducted to contribute to the net-zero emission goal from the energy sector.

In this work, the role of the AI algorithms in deriving the insight of industrial-scale and complex multivariate steam turbine system’s operation (660 MW capacity), sensitivity analysis, optimization of its isentropic efficiency, and the subsequent reduction in emissions as a result of the improved operation of the power plant is presented that addresses the research gap as identified above. A general framework comprising the data collection and visualization, AI-based model development and validation, model-based sensitivity, and its deployment in the optimization environment for the optimization analysis under various operating modes of the steam turbine is presented.

The operational data of the industrial-scale energy device is not only utilized for the AI model development and optimizing the isentropic efficiency of the HP turbine but the bottom-up AI-based modeling approach is extended to calculate the enterprise-level performance indicator, i.e., reduction in the emission load to the environment. This is the novel aspect of this study in deploying the AI models to not only optimize the operation of industrial-scale energy devices but also reduce the emission footprint from the coal power plant for supporting the net-zero goal from the energy sector. The proposed approach is a high innovation considering the general framework development for utilizing the AI modeling tools and optimization algorithms from the viewpoint of contribution toward carbon neutrality for energy-intensive industries^54,55^ by a bottom-up approach, which adds to the main novel aspects of this work.

## 2 Objectives and Methods

**Figure 1:**
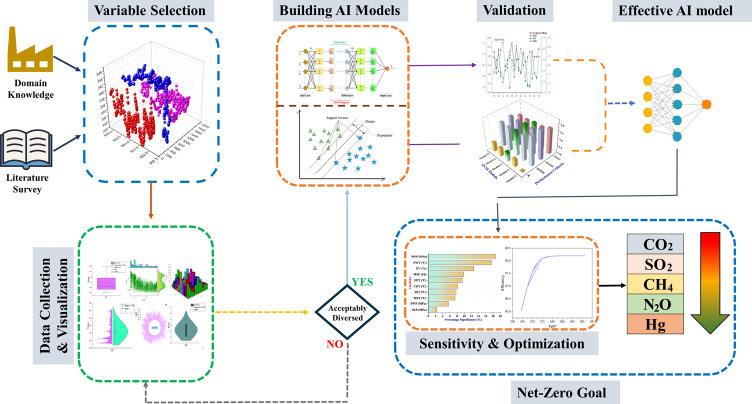
Proposed methodology followed in the research to contribute to the net-zero goal.

The proposed methodology incorporating the AI models for improving the isentropic efficiency of a high-pressure steam turbine and subsequently contributing to the net-zero goal is illustrated in Figure 1. The steam turbine is installed at a 660 MW supercritical power plant, which is equipped with a once-through and π-shaped pulverized boiler, a tangential firing coal combustion technology, a one-steam reheat system, and a single-shaft mounted steam turbine system. A feed water regenerative heating system comprising seven steam heaters and a deaerator is also installed. The advanced sensory network is implanted at various points for measuring different properties of operating parameters, and the measured data is stored in the Supervisory Information System (SIS) of the power plant. The establishment of SIS has numerous advantages in control, data inspection, and management.

For the reliable performance of the AI models, input parameters are chosen carefully based on the domain knowledge of the power plant and the literature review as explained in Section 2.1. The data of the selected parameters is collected from the SIS portal of the Sahiwal Coal Power plant. Subsequently, the data distribution in the output and input spaces is visualized to confirm its suitability for the AI model development. Else, new data sets are to be drawn and make sure that its distribution space in the operating range is reasonable.

ANN and SVM models as bottom-up approaches for modeling the isentropic efficiency of HP steam turbine are selected. The two modeling techniques have presented useful results in scientific and industrial research studies.^56−58^ ANN possesses nonlinear learning characteristics desirable for approximating an ill-defined and complex objective function, which is modeled on the hyperdimensional and interacting input parameters.^59^ On the other hand, SVM has demonstrated an excellent generalization ability in numerous applications ranging from atomic domain to enterprise-level optimization.^26,60^ The performance comparison of the two models is investigated so that a better-performing model could be selected. For this purpose, the external validation data set, primarily unseen during the model’s development, is deployed to be predicted by ANN and SVM. Four statistical measures, namely, correlation coefficient (*R*), root-mean-square error (RMSE), mean absolute percentage error (MAPE), and mean absolute error (MAE), are introduced to define the evaluation criteria. Subsequently, the model showing a better predictive performance in the external validation test is selected. Monte Carlo technique-based sensitivity analysis is carried out to determine the percentage significance of the input parameters toward the HP turbine efficiency. The effects of the important operating parameters on the HP turbine efficiency are studied by the selected AI model. The operating scenarios built on the optimal values of the input parameters (parametric study-based optimization)^19,31^ under the three power production capacities of the power plant, i.e., half-load, mid-load, and full load, are constructed and simulated by the AI model. The operating scenarios are built after extensive consultation with a multidisciplinary team of the power plant’s operation and performance engineers working in various departments. Furthermore, the developed AI model is embedded into the rigorous optimization environment to estimate the operating values of the input parameters corresponding to the maximum HP turbine efficiency. For this purpose, nonlinear programming (NLP) optimization technique is deployed considering the nonlinear nature of the objective function and the input parameters. The results provided by the two optimization techniques are compared. Subsequently, the optimal HP turbine efficiency for the three power generation modes of the power plant is compared with those simulated on the average values of the operating parameters, and the change in the HP turbine efficiency is computed. Moreover, the fuel consumption rate and emissions (CO_2_, SO_2_, CH_4_, N_2_O, and Hg) discharged to the environment corresponding to the improved HP turbine efficiency are also investigated. The proposed framework incorporating the AI model and the optimization technique is deployed to analyze the HP turbine efficiency in order to investigate the improvement in energy efficiency and reduction in emission load to make contribution to net-zero from the energy sector.

### 2.1 Variable Selection and Data Visualization

The fuel combustion system, turbines, and reheating systems are the key energy devices of a power plant. Hundreds of parameters, along with their operational data, are stored in the SIS portal of the power plant. Some of these parameters are controllable by the operator, while a few are adjusted according to the power generation mode of the power complex.

The selection of input parameters is based on the literature review^33,59,61−66^ and the recommendations of the power plant operation and performance engineers. The steam conditions at the inlet of HP turbine have a significant impact on the turbine efficiency. Similarly, the steam exhaust conditions and parameters associated with the steam extractions also have influence on the HP turbine efficiency.^67^ Therefore, the input parameters selected to model the HP turbine efficiency are as follows: main steam temperature (MST), feed water temperature (FWT), feed water pressure (FWP), main steam pressure (MSP), second extraction pressure (SEP), second extraction temperature (SET), governing valve opening (GV), cold reheat temperature (CRT), main steam flow rate (MSF), and first extraction temperature (FET). The output parameter is the HP isentropic turbine efficiency (called HP turbine efficiency) to be modeled on the identified input parameters. The considered parameters are shown in Figure S3 included in the Supporting Information, and the schematic diagram of the power plant along with its description is also provided therein; MST, MSP, GV, and MSF are the operating parameters corresponding to steam conditions at the inlet of HP turbine, whereas CRT is the temperature measured at the exhaust of the HP turbine; FWP and FWT are the conditions of the feed water at the entrance of boiler and are maintained under the integrated operation of steam heaters working on the steam extractions. The steam extraction parameters considered on the suggestions of performance engineers are FET, SET, and SEP that have an impact on the HP turbine efficiency.

The industrial data corresponding to the selected parameters may possess faulty and erroneous observations due to malfunctioning, improper calibration, and maintenance of the sensor that are excluded in the collected data set following the procedure as described in ref (68). Thus, a total of 22 561 data points of all of the parameters are collected from the history of power plant operation during which load is changed from 50 to 100% capacity of the power plant. The statistics of all of the input and output parameters are presented in Table 1. Minimum (Min), mean, median, maximum (Max), standard deviation (S.D.), coefficient of variance (COV), skewness for the data of the parameters, and uncertainty involved in the data measurement by the sensors are presented. The data distribution range of the parameters is reasonably wide as expressed through the max—min value along with S.D. and COV that is beneficial to develop a flexible AI model that is capable of predicting the HP turbine efficiency under different power generation modes of the plant. Furthermore, there also exists a certain degree of skewness in the data that is quite a typical characteristic of the industrial data and is governed by the different modes of the operations of the industrial system. The uncertainty value associated with the data measurements of the sensors is provided by the manufacturer that is reasonably small, thereby indicating the reliability of the measurements collected from the SIS system.

**Table 1:** Statistics of Input and Output Parameters

parameters	min	mean	median	max	S.D.	COV	skewness	uncertainty
main stream temperature (°C)	550	562	563	570	4.6	0.8	–0.4	±1.5 °C
feed water temperature (°C)	263	276	273	298	10.4	1.7	0.5	±1.5 °C
feed water pressure (MPa)	16.0	20.8	19.6	29.2	4.0	22.4	0.7	±0.04%
secondary extraction temperature (°C)	324	334	333	344	5.0	1.4	0.2	±1.5 °C
main stream pressure (MPa)	13.7	17.4	16.5	24.3	3.2	26.7	0.6	±0.04%
cold reheat temperature (°C)	328	337	336	348	4.7	1.4	0.2	±1.5 °C
governing valve opening (%)	23	32.1	30.8	99.9	7.7	14.5	5.5	±0.02%
main stream flow rate (t/h)	1012	1334	1253	1986	268	0.3	0.7	±0.04%
first extraction temperature (°C)	383	395	394	406	5.6	1.2	0.1	±1.5 °C
second extraction pressure (MPa)	2.8	3.6	3.4	5.1	0.7	129.6	0.7	±0.04%
HP turbine efficiency (%)	82.87	86.74	86.79	88.20	0.77	5.37	–0.59	±0.01%

**Figure 2:**
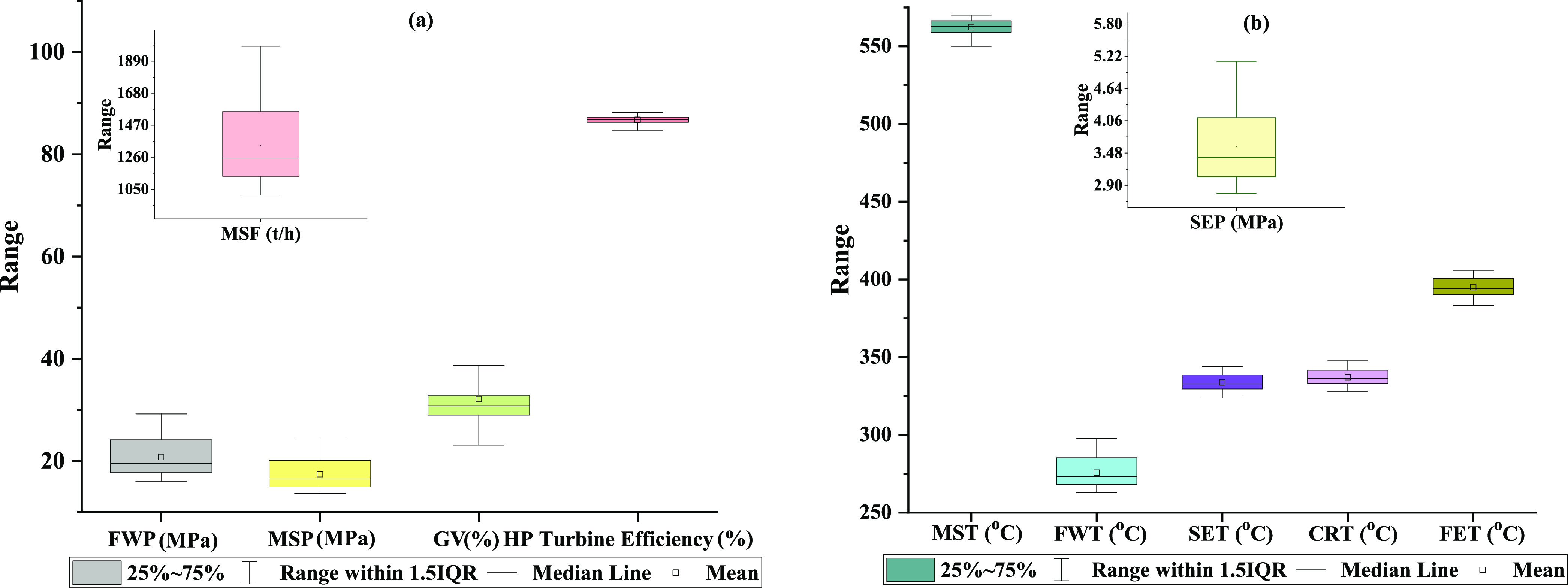
Box plots of the operating parameters. (a) Feed water pressure (FWP), main steam pressure (MSP), governing valve opening (GV), main steam flow rate (MSF), and HP turbine efficiency. (b) Main steam temperature (MST), feed water temperature (FWT), second extraction temperature (SET), cold reheat temperature (CRT), first extraction temperature (FET), and second extraction pressure (SEP).

Moreover, the data distribution of the input and output parameters in the form of box plots is illustrated in Figure 2a,b. Figure 2a depicts the data distribution of five parameters, i.e., FWP, MSP, GV, MSF, and HP turbine efficiency, whereas the input space of MST, FWT, SET, CRT, FET, and SEP is presented in Figure 2b. The box in Figure 2 represents the 25–75% range of data for the input parameters. Moreover, the mean and median values for the parameters are also shown in Figure 2 by point and line, respectively, which are mentioned in Table 1. The mean value is placed nearly at the middle of box plots of almost all of the parameters, demonstrating that the data is well-distributed over the distribution space of all of the parameters.

**Figure 3:**
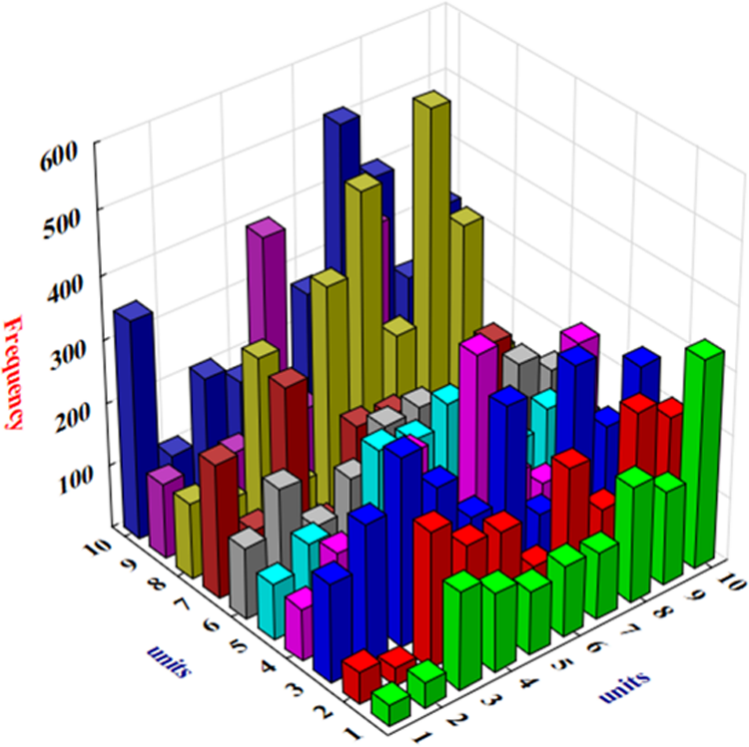
Self-organizing feature map.

A self-organizing feature map (SOFM) is a dimension compression technique to help visualize the distribution of hyperdimensional data in a three-dimensional environment. This technique uses unsupervised learning to present the data visualization with reduced dimensions having the same topology.^69^ The self-organizing map constructed on the input parameters’ data is shown in Figure 3. Reasonable data distribution is observed on the two-dimensional output layer of the constructed SOFM, which confirms that significant input parameters and the collected data associated with them are well-distributed on their operating ranges that are essentially required for building effective AI models.

### 2.2 Artificial Neural Network

Artificial neural network (ANN) is one of the advanced algorithms of AI used for classification and function approximation problems.^25,57,70−72^ ANN is a reliable and competitive mode of a learning algorithm for nonlinear objective functions in comparison with the conventional statistical models.^73^

ANN is a biological form of distributed computation that consists of simple processing nodes and strands. The effect of one unit on the other can be determined by the weight connection between the nodes.^74−76^ The architecture of ANN generally constructed for function approximation problems is presented in the Supporting Information. The input layer consists of neurons equal to the number of input parameters and receives data on which the neural network will learn, organize, or process the information. A hidden layer is present between the input and output layers. ANN processes the data received from the input layer and transmits it to the output layer for further processing. In the hidden and output layers, fundamental data computations occur. The two layers are fully connected to each other, and every node or neuron in the layers refers to a parallel computational element. The information processing at different layers of ANN operation is explained in the following.

At the input layer, different weight values are randomly initiated and assigned to input parameters (*X*_*i*_ = 1, 2, 3, ···, *p*). At the hidden layer, the weights are multiplied with the input vectors constructed on the observations of the input parameters. Subsequently, the summation of the dot product between the weight and input vectors is computed along with the bias value initiated at the hidden layer. The processed information is transformed at each neuron of the hidden layer by the activation function.^73,77^

The hidden layer transmits the processed information to the output layer. At the output layer, further information processing occurs. The weights are randomly initiated at the output layer, multiplied with the received hidden layer’s information, and the summation is calculated along with the bias value. The activation function transforms the information, and the output value is calculated at the output layer. A general mathematical expression of ANN working is given as
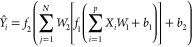
1here, *Ŷ*_*i*_ is the ANN-predicted output response corresponding to the input vector *X*_*i*_. *W*_1_ and *W*_2_ are the weights, *f*_1_ and *f*_2_ are the activation functions, *b*_1_ and *b*_2_ are the bias values that are imposed on the hidden and output layers of ANN, respectively.

The training algorithm executes in an iterative process, which attempts to minimize the mean-squared errors (*M*) to optimize the training process
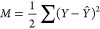
2where *Y* represents the actual output value and *Ŷ* refers to the model-simulated output value in the feed forward training of ANN. Generally, a large number of iterations are to be executed for constructing an ANN model and to minimize *M*. In each iteration, *M* is calculated and the error signal is propagated backward to the network. The training algorithm adjusts the weights applied at the hidden and output layers as
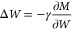
3where γ is the learning rate that defines the step size while moving toward the minimization of the loss function. *M* is computed corresponding to the updated weights, and the error reduction loop continues until the stopping criterion is achieved, i.e., maximum number of epochs are completed, the change in error gradient is less than the threshold value, and maximum validation failure counts are observed.^78^

In this work, a data split ratio of 0.8, 0.1, and 0.1 is used for data allocation to training, testing, and validation data set. The number of neuron in the hidden layer is an important hyperparameter to be tuned rigorously to achieve the excellent predictive and generalization performance of the ANN model. The number of hidden layer neurons act as a feature detector to mine the underlying information in the data. It also controls the complexity introduced in the algorithm to approximate the given function space. The number of hidden layer neurons are selected on a hit-and-trial basis. However, our group has found that the optimal number of hidden layer neurons can be from 1*×* to 3*×* of the input layer neurons. Thus, hidden layer neurons are varied from 10 to 30 in this work. Another important hyperparameter to be optimized is the number of hidden layers. It is provided in the literature that a single hidden layer ANN can approximate the nonlinear function with good accuracy given that enough number of hidden layer neurons are provided.^79^ Therefore, a shallow three-layered ANN architecture is initiated in this work to model the HP turbine efficiency. The activation function applied on the hidden and output layers is tangent hyperbolic and linear, respectively. Gradient descent with momentum as a training algorithm is utilized for the parametric optimization of the ANNs,^17,18^ and the learning rate of 0.01 is used. MATLAB 2021a is used for the training of the ANN model. The developed ANN models on different architectural configurations are retained, and their comparative prediction performance on the unseen external validation data set is evaluated as described in Section 3.1.2 in order to investigate their predictive and generalization performance.

### 2.3 Support Vector Machine

Support vector machine (SVM) is a supervised learning technique that has been introduced to solve problems related to classification and regression-based problems. The structural risk minimization principle presented by Vapnik is used to reduce the generalization error and is implemented for training the SVM models. In SVM, a single or multiple hyperplanes are used in higher-dimensional space and data points are classed using the hyperplanes.^80^ The data points on one side of the hyperplane are classified in separate group than the data on the other side. These hyperplanes are then used for the regression and classification tasks.^81^ The details regarding the data segregation by the hyperplane are provided in the Supporting Information (refer to Figure S5).

The training data set for developing an SVM model is given as
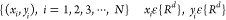
4where *x*_*i*_ are the data points from the input space of the parameters, *y*_*i*_ is the corresponding output, and *N* is the size of the data set. The linear SVM models in primal and dual forms cannot develop the optimal solutions for nonlinear objective functions. The Lagrangian function is introduced to apply the SVM algorithm for estimating the optimal solutions for the nonlinear functions. Non-negative numbers, i.e., α_*n*_ and α_n_^*^, are introduced for each observation of the data set. Thus, the loss function for the dual form of nonlinear SVM is given as
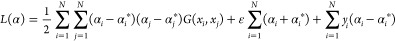
5subject to the constraints
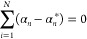
6
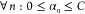
7
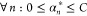
8where, *G*(.,.) is the kernel function and ε is the epsilon margin around the hyperplane. Similarly, the box constraint is given by *C*, which is the penalty factor imposed on the observations violating the ε-intensive boundaries. It also determines the trade-off between the model predictive accuracy and the amount up to which deviations larger than ε are tolerated.

The Karush–Kuhn–Tucker (KKT) complementarity conditions are the optimization constraints that are essentially implemented for developing the optimal solution of the nonlinear dual problem of the SVM. These optimization constraints are given as
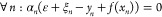
9
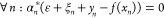
10
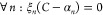
11
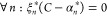
12Here, ξ and ξ* are the slack parameters introduced to tolerate the deviations beyond the ε-tube.

The final SVM function derived for predicting the observations of the input parameters is given as
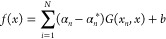
13here, *x*_*n*_ are the support vectors and *b* is the bias value. The kernel function is of three types, namely, linear, polynomial, and Gaussian. Usually, the Gaussian-type kernel function is used for projecting the hyperdimensional input data into space where optimization constraints 6–12 applied to the objective function contribute to determining the optimal solution of the objective function.

MATLAB 2021a is used in this work for training the SVM model. Bayesian optimizer, acquisition function, and expected improvement per second function are incorporated for tuning the hyperparameters like box constrain (*C*) and Epsilon (ε). The details regarding the hyperparameter tuning and the error convergence are provided in the Supporting Information (refer to Figure S6).

## 3 Results and Discussion

### 3.1 Evaluation of AI Models

The developed AI models were evaluated based on their predictive performance, which are described in detail in the following section.

#### 3.1.1 Evaluation Criteria

After training the SVM and ANN on the operational data, there is a need to establish an evaluation criterion for the selection of the better-performing AI model. Four statistical parameters, namely, correlation coefficient (*R*), root-mean-square error (RMSE), mean absolute percentage error (MAPE), and mean absolute error (MAE),^17^ are selected to evaluate the predictive performance of the trained AI models. The mathematical expression of the statistical parameters is as follows
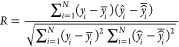
14
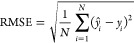
15
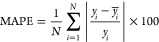
16
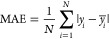
17here, *N* represents the sample size, while *y*_*i*_, *y̅*_*i*_, *ŷ*_*i*_, and  are the actual values, the mean actual values, the predicted values, and the mean predicted values, respectively. *R* ranges from −1 (negatively correlated) to 1 (positively correlated), whereas *R* = 0 represents that there is no relationship between the predicted and actual values. On the other hand, RMSE, MAPE, and MAE are the error terms computed to gauge the deviation in the model-predicted responses with the actual values and should be made as low as possible, thereby indicating the good modeling and predictive performance of the developed AI models.

#### 3.1.2 External Validation of the Trained AI Models

The prediction efficiency of the trained AI models, i.e., ANN and SVM, is evaluated on the defined evaluation criteria. The AI models are externally validated on the unseen data that was not deployed for the training of ANN and SVM models. The external validation data set has 848 random observations, each having operating values for all input and output parameters. Moreover, it is ensured that the data points are well dispersed on the range of the operating parameters; thus, the predictive performance of the models is evaluated in different operating regions of the input parameters.

**Figure 4:**
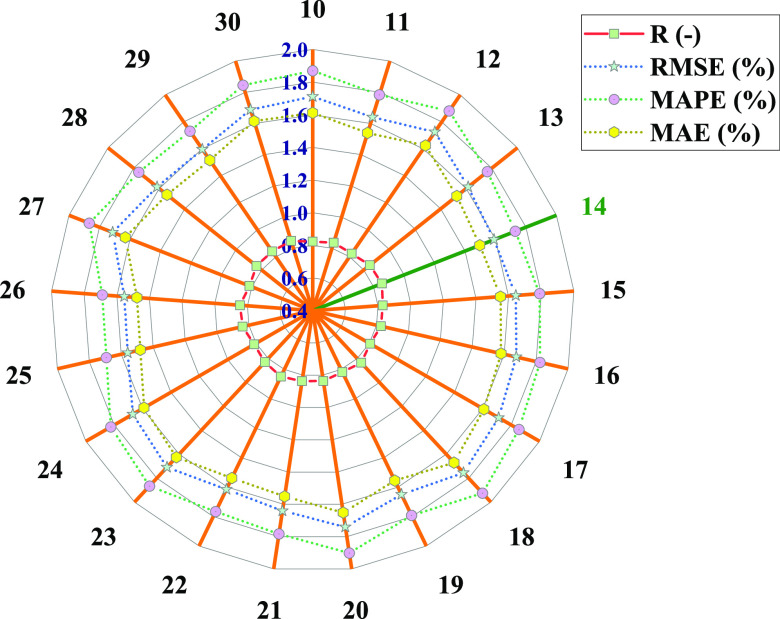
*R*, RMSE, MAPE, and MAE computed for ANN having hidden layer neurons from 10 to 30.

ANN models with 10–30 neurons in the hidden layer are developed, and the prediction efficacy of the models is checked by an external validation test. The models’ predicted responses are compared with the actual values of the HP turbine efficiency and the statistical terms included in the evaluation criteria, i.e., *R*, RMSE, MAPE, and MAE are computed. As shown in Figure 4, the ANN model with 14 neurons in the hidden layer has presented the highest *R*-value (0.85), whereas RMSE, MAPE, and MAE are also comparatively minimum, i.e., 1.59, 1.73, and 1.50%, respectively, for the same network in comparison with that of other ANN models. Therefore, ANN with 14 neurons in the hidden layer is chosen out of the trained ANNs.

**Figure 5:**
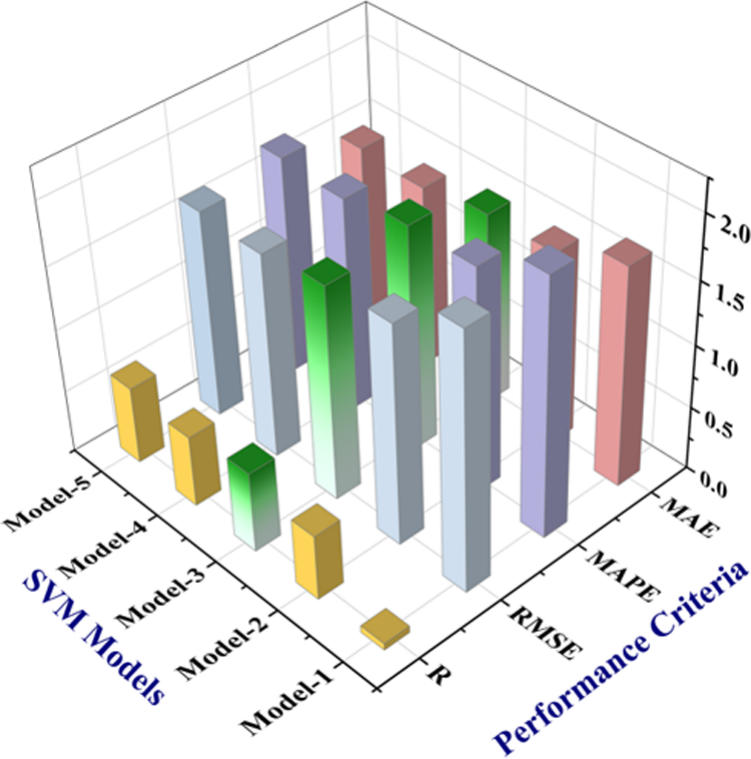
Comparative prediction performance of five SVM models on R, RMSE, MAE, and MAPE.

Five optimizable SVM models with the Gaussian kernel function are trained subjected to rigorous hyperparameter tuning, and their prediction performance for the external validation test is also measured by *R*, RMSE, MAPE, and MAE values, as shown in Figure 5. It is found that SVM model 3 has the highest value of *R* measuring 0.64, and its error values (RMSE = 1.69%, MAPE = 1.79%, MAE = 1.53%) are comparatively lower than that of other SVM models. It demonstrates the comparatively better predictive and generalization performance of the SVM model to predict the external validation data set. Therefore, SVM model 3 is selected out of the trained SVM models for conducting subsequent analysis.

#### 3.1.3 Performance Comparison between ANN and SVM Models

The performance comparison of the two AI models in predicting the external validation data set is made to select a better AI model for the HP turbine efficiency. Table 2 presents the statistical measures computed on the external validation data set’s prediction by the best-performing ANN and SVM models. *R*-value, RMSE, MAE, and MAPE for ANN are 0.853, 1.59, 1.50, and 1.73%, respectively, which are comparatively better than that of SVM, i.e., 0.64, 1.70, 0.02, and 1.53%, respectively. Table 2 clearly shows that ANN has performed comparatively well in simulating the external validation data set, thereby ensuring the good functional mapping developed between the input and output parameters. Therefore, ANN with 14 neurons in the hidden layer is chosen for further analysis, as presented in the next section.

**Table 2:** Prediction Performance Comparison of Selected ANN and SVM Models

AI Model	*R* (−)	RMSE (%)	MAPE (%)	MAE (%)
ANN	0.85	1.59	1.73	1.50
SVM	0.64	1.69	1.79	1.53

The better predictive performance of ANN is attributed to its suitability for the system-level operational modeling where a complex network comprising a large number of operating parameters is present. In modeling such a nonlinear and quantitative nature of the objective function built on the hyperdimensional input space, the backpropagation algorithm works well to approximate the system.^17^ System- and component-level problems of industrial-scale production facilities are continuous data functional approximation problems.^19,25,82^ On top of that, large industrial complexes generate control data for which established function approximators like backpropagation-based fully connected multilayer perceptron models would perform better.^56^ Such machine learning algorithms, which are fundamentally and architecturally classifiers (like SVM), and their modified variants for regression-based learning algorithms cannot perform on par with ANN for component/system-level complexity and nonlinearity.^17−19^

### 3.2 Sensitivity Analysis

**Figure 6:**
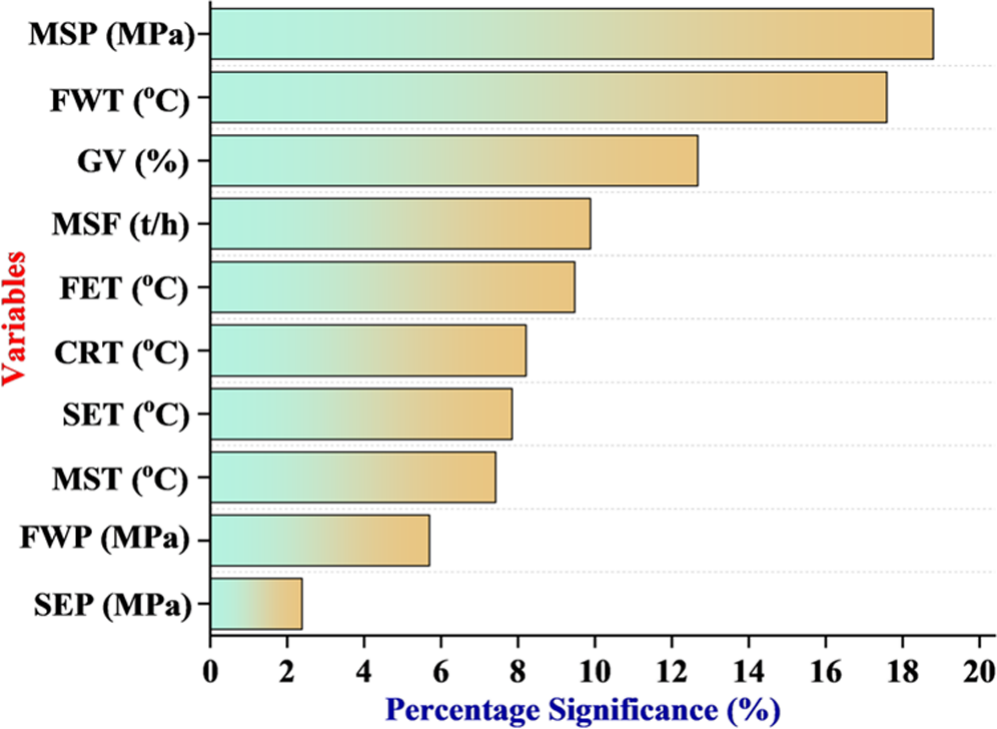
Monte Carlo-based sensitivity analysis of the input parameters toward the prediction of HP turbine efficiency.

Monte Carlo technique-based sensitivity analysis is carried out to investigate the percentage significance of the input parameters toward the HP turbine efficiency. The detailed procedure for constructing the Monte Carlo experiments and carrying out the sensitivity analysis is described in our earlier reported research.^18,19^Figure 6 shows the percentage significance of the input parameters to predict the HP turbine efficiency. MSP and FWT are found to be the first two input parameters having the percentage significance value of 18.8, 17.6, and 12.7%, respectively. The least significant parameter is observed to be SEP having a percentage significance value of 2.4%. MSP is the pressure of the main steam before the HP turbine and presents the work potential of the steam for power generation. Thus, it has a significant and positive impact on the HP turbine efficiency. Similarly, FWT is the temperature of the feed water before entering the boiler and it is maintained under the synchronized and integrated operation of HP heaters working on the steam turbine extraction, thereby having a significant and positive impact on the HP turbine efficiency. GV is the percentage opening of the governing valve and thus controls the amount of the steam flow entering the HP turbine and is termed to be the third most significant input parameter having a percentage significance value of 12.7%. MSF is the flow rate of the main steam that expands in the multistage HP steam turbine and contributes a percentage significance of 9.9% to predict the HP turbine efficiency and has a positive impact on the turbine efficiency. Similarly, the percentage significance values of FET, CET, SET, MST, and FWP are as follows: 9.5, 8.2, 7.8, 7.4, and 5.7%, respectively.

### 3.3 Parametric Study and Simulating the Operating Scenarios

The impact of important operating parameters on the HP turbine efficiency is studied using the developed ANN model. The experiments, including individual or pair of operating parameters at the three power generation modes of the power plant, i.e., half-load, mid-load, and full load, are constructed and predicted by the model under the supervision of the power plant engineers. Furthermore, the optimal operating scenarios comprising the influence of almost all operating parameters are also constructed, simulated by the developed ANN model, and the HP turbine efficiency gain is compared with that of the average-controlled values of operating parameters under the sustained power generation. The details associated with the parametric study and operating scenarios are presented in the subsequent subsections.

#### 3.3.1 Effect of Main Steam Temperature on the HP turbine efficiency

Main steam temperature is one of the critically controlled operating parameters at steam power plants. The temperature is maintained in its operating range, ensuring the effective operation control of heating surfaces and devices installed in the boiler. The HP turbine efficiency is significantly affected by the main steam temperature during the power generation operation. Moreover, the overall efficiency of the power plant is also affected by the main steam temperature; thereby, it has technoeconomic implications on power production.^18^

**Figure 7:**
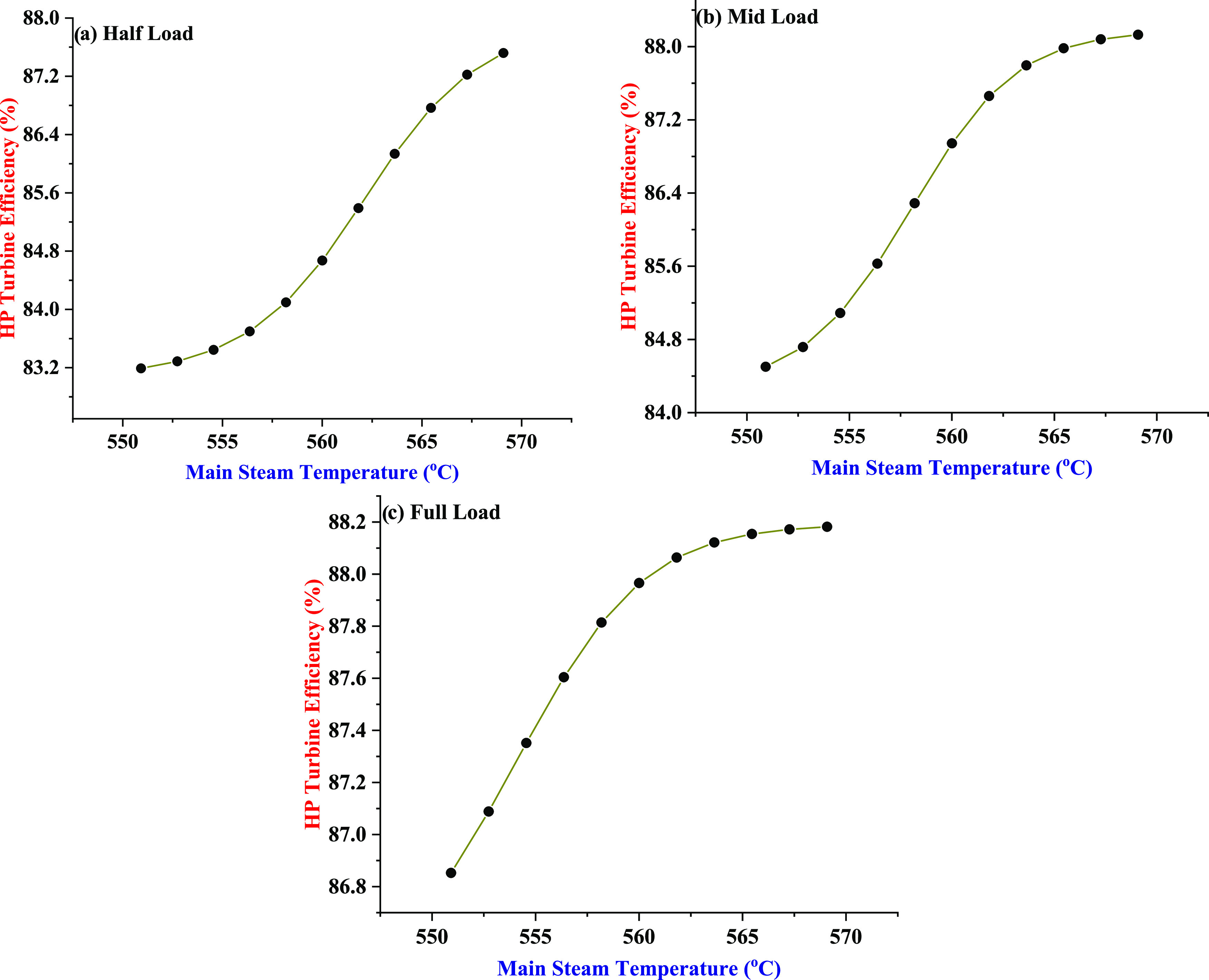
Effect of the main steam temperature on the HP turbine efficiency under three power generation capacities of the power plant: (a) half-load, (b) mid-load, and (c) full load.

The effect of main steam temperature on the HP turbine efficiency is studied under three power generation capacities of the power plant, i.e., half-load, mid-load, and full load. The operating range of the temperature is selected as provided by the manufacturer of the HP steam turbine. The impact of main steam temperature on HP turbine efficiency at the half-load, mid-load, and full-load scenarios of the power production is presented in Figure 7a–c, respectively. A general increasing trend in the HP turbine efficiency is observed when the main steam temperature is increased from 550 to 570 °C. It is noted that every 10 °C increase in the main steam temperature drives the HP turbine efficiency up, on an average, by 2.57, 2.13, and 0.76% corresponding to half-load, mid-load, and full-load operating modes of the power plant respectively. The increase in the main steam temperature makes higher work potential available at the inlet of the turbine, which is effectively utilized by steam expansion in the multistage HP turbine, and therefore, HP turbine efficiency increases.^67^ The findings can be useful for the power and process industry community generating power from steam turbine for effectively maintaining the operating values of the main steam temperature that can enhance the HP turbine efficiency.

#### 3.3.2 Effect of Feed Water Temperature on the HP Turbine Efficiency

Feed water temperature is also among the power plant operation control parameters that are critically maintained in their limiting operating ranges during the power generation operation. The temperature is controlled by the regenerative heating system, which consists of a number of steam heaters installed for heating up the feed water. The steam extractions taken from the steam turbines are passed through the steam heaters for the feed water heating purpose. Thus, the preheating of feed water reduces the thermal load of the boiler for producing the main steam at the rated parameters, and the overall efficiency of the power plant is improved.^18^ It is important to mention here that the operating window of the feed water temperature measured at the inlet of the boiler is very narrow corresponding to the sustained power production since it greatly influences the fuel consumption rate and thermal energy spent for the power production.

**Figure 8:**
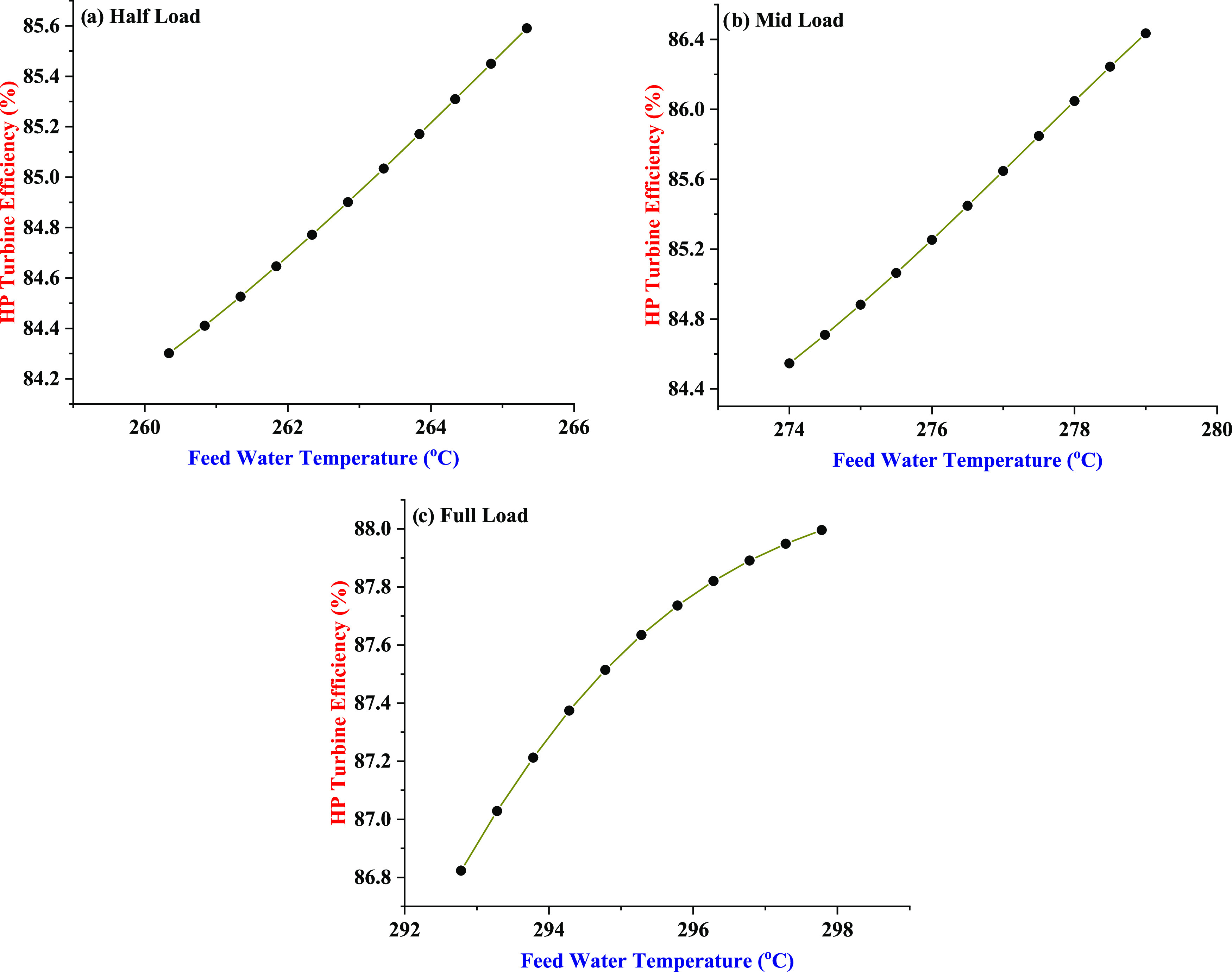
Effect of feed water temperature on the HP turbine efficiency under three power generation capacities of the power plant: (a) half-load, (b) mid-load, and (c) full load.

Figure 8a–c presents the impact of feed water temperature on the HP turbine efficiency at half-load, mid-load, and full-load capacities of the power plant. A general increasing trend in the HP turbine efficiency is observed with the increase in feed water temperature in its operating range as selected on the basis of the sustained power production mode of the power plant. It is estimated that the HP turbine efficiency is increased, on an average, by 0.76, 1.11, and 0.67% for every 3 °C increase in feed water temperature. The increase in HP turbine efficiency also complies with the operational physics of the power plant considering its significance toward the overall thermal efficiency and power generation operation of the plant. The improvement in HP turbine efficiency driven by the increase in feed water temperature suggests the effective heat transfer and better operational control of the HP heaters toward feed water heating typically installed in the power plants. Therefore, the steam extraction process for the feed water heating appears to be improved that does not only provide the improved performance to maintain the feed water temperature corresponding to the sustained power generation but also the HP turbine efficiency is also improved.

#### 3.3.3 Simultaneous Effect of the Governing Valve Opening and Main Steam Pressure on the HP Turbine Efficiency

**Figure 9:**
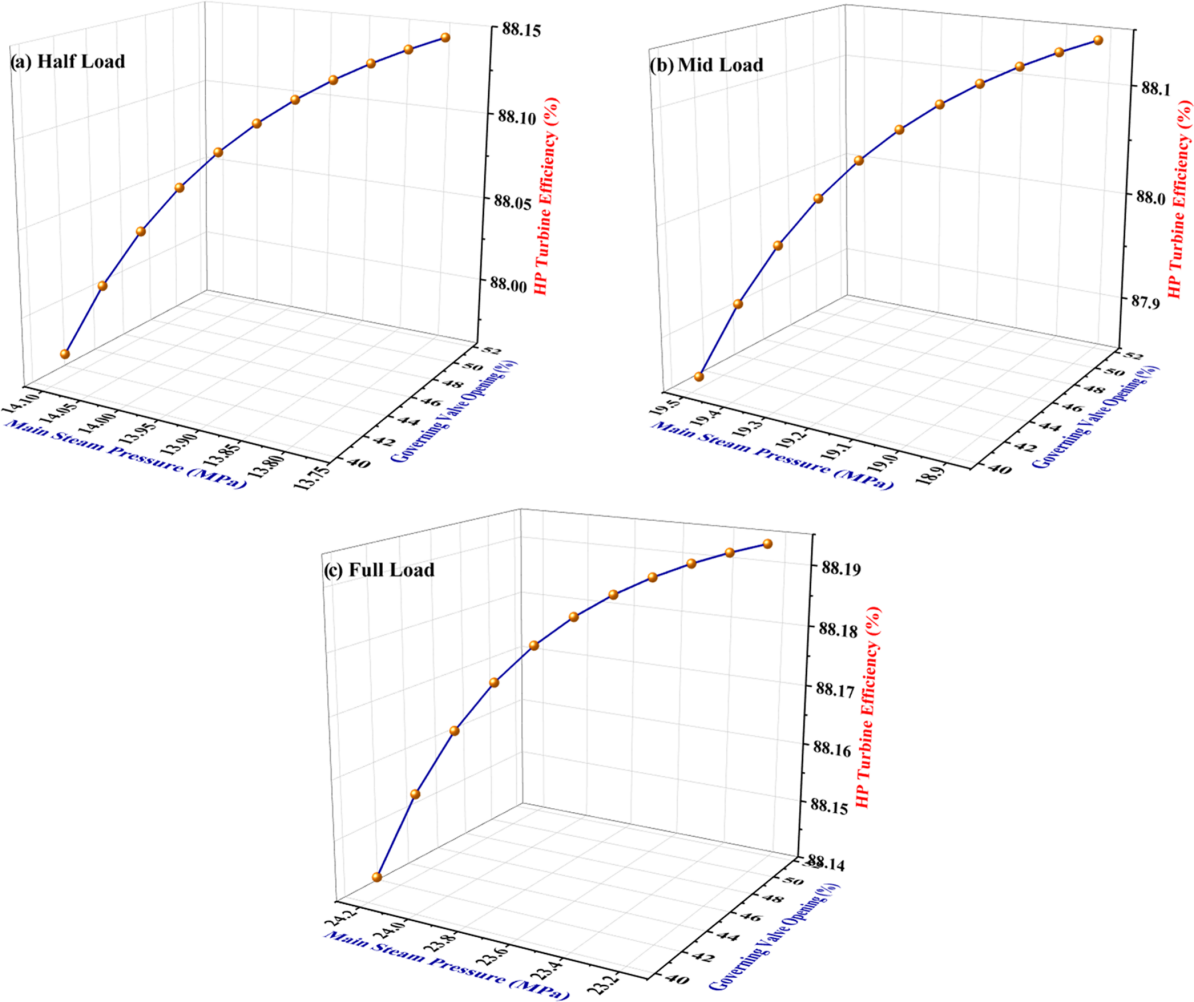
Effect of governing valve opening and main steam pressure on the HP turbine efficiency under three power generation capacities of the power plant: (a) half-load, (b) mid-load, and (c) full load.

The governing valve is mounted on the main steam pipeline connected to the HP turbine. The opening of the governing valve results in increased steam flow to the HP turbine for the same thermal conditions of the steam. However, governing valve opening is simultaneously adjusted at the same load by marginal variation in the main steam pressure to ensure a nearly constant main steam flow. Thus, the combined effect of governing valve opening and the marginal variation in the main steam pressure is investigated in the developed ANN model. Figure 9a–c refers to the governing valve opening from 41 to 51% with the marginal variation in the main steam pressure at three power generation capacities of the power plant, and the combined effect of these two parameters on the HP turbine efficiency is presented. A nominal increase in the HP turbine efficiency is observed as a result of the simultaneous governing valve opening and a slight decrease in the main steam pressure for three power generation states of the power plant. It is found that, on an average, the HP turbine efficiency is increased by 0.21, 0.26, and 0.03% for every 5% opening of the governing valve and every decrement in the main steam pressure by 0.14, 0.27, and 0.44 MPa at half-load, mid-load, and full-load state of the power plant, respectively. Thus, the increase in the governing valve opening within the operational limit corresponding to three power generation modes, i.e., half-load, mid-load, and full load, provides the effective steam conditions that result in improved HP turbine efficiency. Thus, the findings can be helpful to the power sector and process industries generating power from steam turbine and can maintain the high energy efficiency of the HP turbine.

#### 3.3.4 Comparison of the Parametric Study and NLP-Based Optimization with the Operation of the HP Turbine under Three Operating Scenarios

The simulated effects of the operating parameters as presented in Sections 3.3.1–3.3.3 provide the basis for selecting their optimal values under parametric study-based optimization.^19^ The operating values of the parameters corresponding to which HP turbine efficiency is maximum under three power generation modes are considered. A similar approach is used in ref (18) for the parametric study-based optimization. Furthermore, the selection of the optimal values for constructing the operating scenarios is also based on the power plant operational physics that complies with the power plant operation.

The NLP optimization analysis is also conducted on optimizing the HP turbine efficiency for three power generation modes of the power plant. The NLP optimization technique is employed considering the nonlinear nature of the objective function and the nonlinear relationships among the input parameters and the objective function. The general mathematical expression of the constrained NLP problem is written as
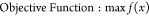
18

subject to
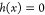
19
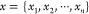
20
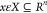
21
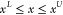
22where *x* is a set of optimization parameters defining the objective function *f*(*x*). The objective function, in this work, is the HP turbine efficiency, and the optimization parameters are the operating parameters as mentioned in Table 1. *h*(*x*) are the equality constraints representing the ANN model developed in Section 2.2.^83,84^*x*^*L*^ and *x*^*U*^ refer to the lower and upper bounds on the input parameters (*x*_1_, *x*_2_, ···, *x*_*n*_), respectively, and the optimal value of the objective function is determined by solving the nonlinear optimization problem described above. The ANN model constructed on the operating parameters for modeling the HP turbine efficiency is incorporated within the NLP problem to maximize the objective function. The objective function is maximized under the bounds of the operating parameters for the three power generation scenarios.

**Figure 10:**
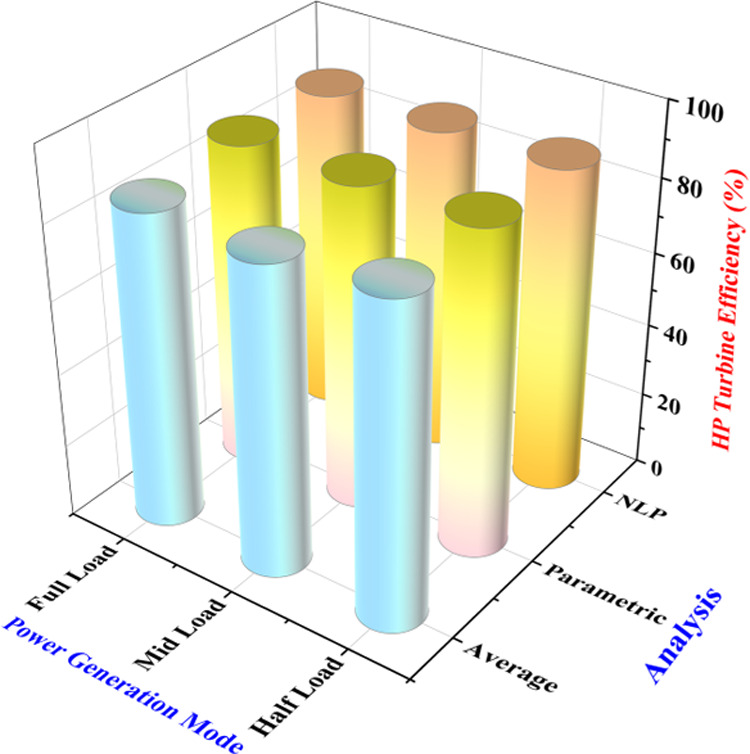
Comparison of HP turbine efficiency corresponding to the average and optimal values of the operating parameters under three power generation capacities of the power plant: (a) half-load, (b) mid-load, and (c) full load.

The optimal values of the HP turbine efficiency for the parametric study and NLP-based optimization are compared, and a good agreement among the optimal solutions of the two optimization techniques is found, i.e., the percentage deviation in the optimal values of the HP turbine efficiency for the two techniques is 0.03, 0.19, and 0.02% for half-load, mid-load, and full-load, respectively. Moreover, the average values of the operating parameters are taken under the three sustained power production modes since the power plant operation is maintained around the average values by the power plant operators. The operating scenarios built on the average values of the operational parameters are simulated, and the HP turbine efficiency is compared with those estimated from the parametric study and NLP-based optimization techniques. The HP turbine efficiency for average settings of the operating parameters, parametric study, and NLP optimization is graphically presented in Figure 10. It is estimated that the HP turbine efficiency, on an average, is increased by 1.43, 5.09, and 3.40% compared to those obtained by simulating the average values of the operating parameters under half-load, mid-load, and full-load capacities of the power plant operation.

#### 3.3.5 Emission Reduction Equivalent of Increment in HP Turbine Efficiency

The emission reduction equivalent is investigated as a result of improvement in HP turbine efficiency achieved through the operating scenarios built on the optimal values of the operational parameters. The fuel consumption rate of the power plant corresponding to the average and optimal values of HP turbine efficiency under three power generation capacities is analyzed. It is estimated that approximately 3.33, 7.05, and 4.04 t/h fuel consumption rates of the power plant are saved compared with the average values of HP turbine efficiency under half-load, mid-load, and full-load operating modes of the power plant, respectively. The fuel savings are credited to the simultaneous improvement in the boiler and turbine efficiencies of the power plant due to the integrated nature of system’s operation. Furthermore, the fuel savings also contribute toward the improved overall efficiency of the power complex.^19^

**Figure 11:**
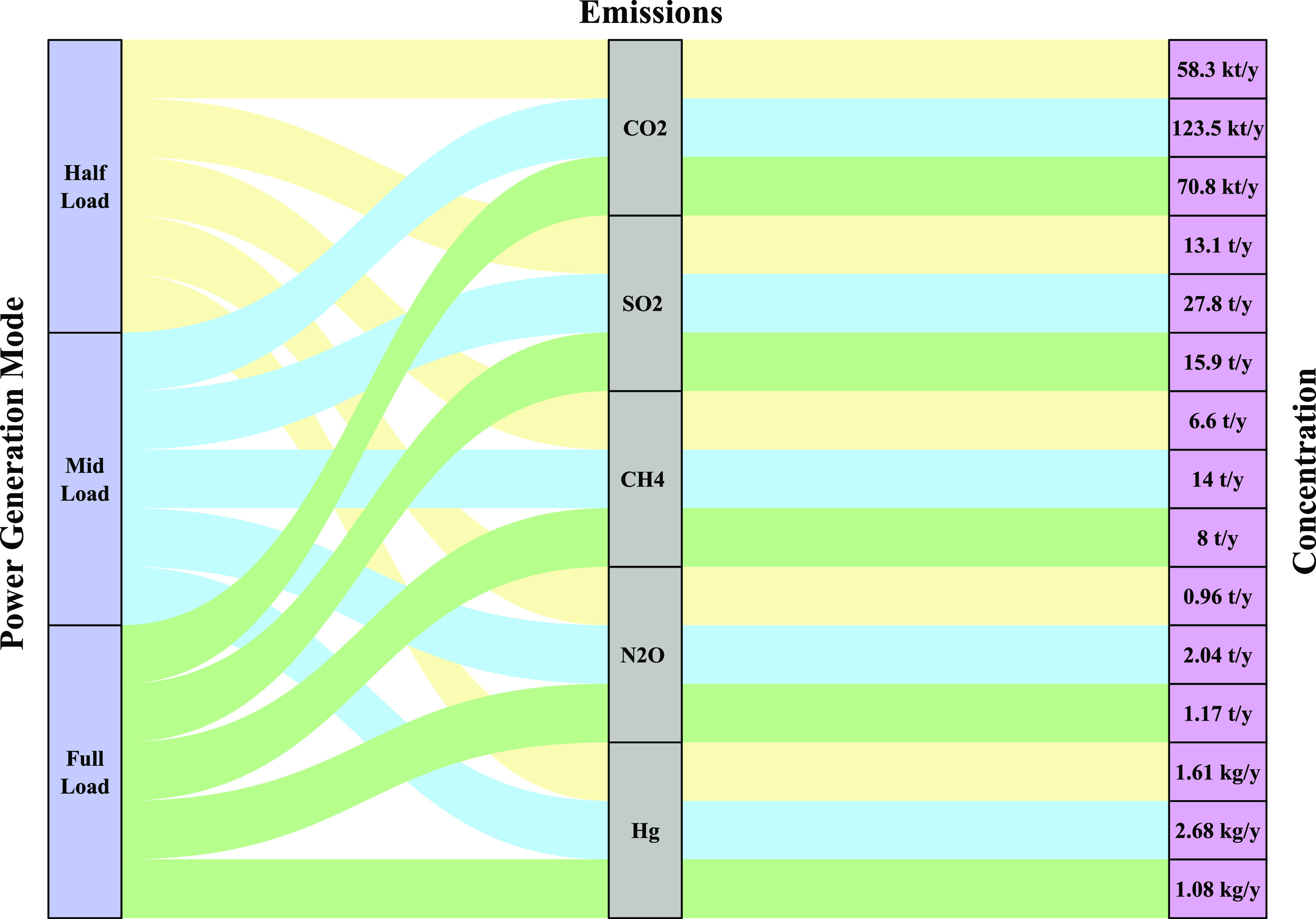
Annual reduction in CO_2_, SO_2_, CH_4_, N_2_O, and Hg emission discharges under half-load, mid-load, and full-load operating modes of the power plant.

The fuel savings are converted into the annual reduction in CO_2_, SO_2_, CH_4_, N_2_O, and Hg emission discharges from the power plant,^19^ as shown in Figure 11. The reduction in emission concentration is calculated for the three power generation modes of the power plant, i.e., half-load, mid-load, and full load. Figure 11 shows an alluvial diagram that connects the concentration value (right-side column) of the emission (middle column) with the power generation mode (left-side column). It is observed that a significant reduction in CO_2_ emission is achieved, measuring 58.3, 123.5, and 70.8 kilo ton/year (kt/y), corresponding to half-load, mid-load, and full-load operating modes of the power plant, respectively. Similarly, a noticeable reduction in SO_2_, CH_4_, and N_2_O emissions, i.e., 13.1, 27.8, and 15.9 t/y, 6.6, 14, and 8 t/y, 0.96, 2.04, and 1.17 t/y, are estimated corresponding to half-load, mid-load, and full-load power generation modes of the plant, respectively, whereas the Hg emission reduction estimated under three power generation capacities of the plant are as follows: 1.61 kg/y for half-load, 2.68 kg/y for mid-load, and 1.08 kg/y for full load.

The AI model-based results presented in this research demonstrate the practical and effective utilization of AI-based modeling and optimization analysis for enhancing the performance of industrial-scale steam turbine that is a fairly large industrial system comprising a hyperdimensional input space. Though the analysis is carried out for the operation optimization of the steam turbine that is a specific class of system-level problem, the proposed AI-based analysis framework can be extended to conduct the performance enhancement of large-scale industrial systems like biomass-based energy systems, petrochemical industries, and manufacturing systems corresponding to component-, system-, and strategic-level operations of industrial system, as described in ref (19). Furthermore, the AI-based industrial analytics can help operation and performance engineers of industrial complexes develop optimal operational practices and strategies for effective operation control and real-time optimization of the energy devices and systems. As a result, the energy-efficient operation management of the energy systems and petrochemical industries can reduce the emission load to the environment and contribute to the global net-zero emission targets for environmental sustainability and industry 4.0 vision of digitalization of industrial systems.

## 4 Conclusions

In this paper, data-driven AI-based model and optimization techniques are deployed to conduct the energy efficiency improvement analysis for an industrial-scale steam turbine. Data on the selected operating parameters is taken from the power plant, and its distribution in the input and output spaces is visualized. Having confirmed the suitability of the data, two AI models, i.e., ANN and SVM, are trained under rigorous hyperparameter tuning. The comparative predictive performance of the AI models for the external validation data set confirmed the better prediction and generalization performance of the ANN model.

Monte Carlo sensitivity analysis is conducted on the trained ANN model to evaluate the significance of the input parameters toward the HP turbine efficiency. MSP, FWT, and GV are termed to be the three most significant parameters having percentage significance values of 18.8, 17.6, and 12.7%, respectively.

The impact of individual and combination of operating parameters under three power generation capacities of the power plant, i.e., half-load, mid-load, and full load on the HP turbine efficiency, is evaluated by the ANN model. It is found that for every 10 and 3 °C increase in the main steam temperature and feed water temperature at half-load, mid-load, and full load, the HP turbine efficiency, on an average, increases by 2.57, 2.13, and 0.76% and 0.76, 1.11, and 0.67%, respectively. Similarly, the simultaneous effect of every 5% increase in the governing valve opening and a nominal decrease in main steam pressure drives the HP turbine efficiency up, on an average, by 0.21, 0.26, and 0.03% under half-load, mid-load, and full-load operating modes of power plant, respectively.

The optimal operating scenarios are constructed based on the best set values of the operating parameters (parametric study-based optimization). Moreover, NLP optimization analysis is also performed for maximizing the turbine efficiency subjected to the operating range of the operating parameters. A good agreement is found among the results of the parametric study and the NLP-based optimization analyses. Furthermore, it is found that HP turbine efficiency is increased by 1.43, 5.09, and 3.40% in comparison with those simulated on the average values of the operating parameters under half-load, mid-load, and full-load capacities of the power plant operation. A significant reduction in CO_2_ emissions measuring 58.3, 123.5, and 70.8 kt/y and noticeable mitigation of SO_2_, CH_4_, N_2_O, and Hg emissions corresponding to half-load, mid-load, and full-load operating modes of the power plant are also estimated as a result of the improved HP turbine efficiency.

The proposed AI-based modeling and optimization framework for obtaining insights into the steam turbine’s operation, optimizing its energy efficiency, and the subsequent reduction in the emission discharge contributes to net-zero goal from the coal power plant. Furthermore, the findings advocate the potential of AI modeling tools to be utilized by industrial managers and big industrial customers like oil and gas, fertilizer and process industries, and fossil- and renewable-based power generation systems to enhance the industrial systems’ performance and contribute to net-zero emissions.

## 5 Future Work

In future studies, it is recommended to conduct the multiobjective optimization under uncertainty for the operation optimization of the steam turbine system, incorporating the heat rate and energy efficiency as the two objectives. Furthermore, the digital twin for the steam turbine operation is recommended to be developed for the smart operation, ensuring the high energy efficiency of energy systems that contributes to the net-zero goal.

## Supporting Information Available

CO_2_ emission and electricity power generation trend in emerging and advanced economies, schematic diagram of power plant operation, architecture of artificial neural network, hyperplane geometry in support vector machine, and hyperparameter tuning for SVM network (PDF)

## Nomenclature

AI: artificial intelligence
ANN: artificial neural network
*b*: bias
*R*: correlation coefficient
HP: high pressure
IEA: International Energy Agency
MAE: mean absolute error
MAPE: mean absolute percentage error
*i*: observations
RMSE: root-mean-square error
*N*: size of data set
SIS: Supervisory Information System
SVM: support vector machine
*w*: weight
