# Supplementary material for: Artificial Intelligence Modeling-Based Optimization of an Industrial-Scale Steam Turbine for Moving toward Net-Zero in the Energy Sector
Source: ACS Omega. 2023 Jun 2;8(24):21709–25. doi: 10.1021/acsomega.3c01227 (PMC10285957; doi:10.1021/acsomega.3c01227)
Supplement: Supplementary file 1 — ao3c01227_si_001.pdf [file ao3c01227_si_001.pdf]

# Artificial intelligence modelling based optimization of an industrial scale steam turbine for moving towards net-zero in the energy sector

Waqar Muhammad Ashraf[1*], Ghulam Moeen Uddin[2], Rasikh Tariq[3], Afaq Ahmed[2], Muhammad Farhan[2], Muhammad Aarif Nazeer[2], Rauf Ul Hassan[2], Ahmad Naeem[4], Hanan Jamil[2], Jaroslaw Krzywanski[5], Marcin Sosnowski[5], Vivek Dua[1*]

[1]*Sargent Centre for Process Systems Engineering, Department of Chemical Engineering, University College London, Torrington Place, London WC1E 7JE, UK*

[2]*Department of Mechanical Engineering, University of Engineering & Technology, Lahore, Punjab 54890, Pakistan*

[3]*Facultad de Ingeniería, Universidad Autónoma de Yucatán, Av. Industrias No Contaminantes por Anillo Periférico Norte, Apdo. Postal 150, Cordemex, Mérida, 97203, Yucatán, México*

[4]*Department of Automotive Engineering Technology, Punjab Tianjin University of Technology, Lahore 54000, Pakistan*

[5]*Faculty of Science and Technology, Jan Dlugosz University in Czestochowa, 13/15 Armii Krajowej Av., 42-200 Czestochowa, Poland*

Corresponding Author: waqar.ashraf.21@ucl.ac.uk; v.dua@ucl.ac.uk

## $CO_2$ emission and electricity power generation trend in emerging and advanced economies

$CO_2$ emissions discharge from the various sources like electricity, industry, transport, building and other sources are estimated by the IEA from 2020 to 2050 for advanced and emerging economies, and are presented in Figure S1.

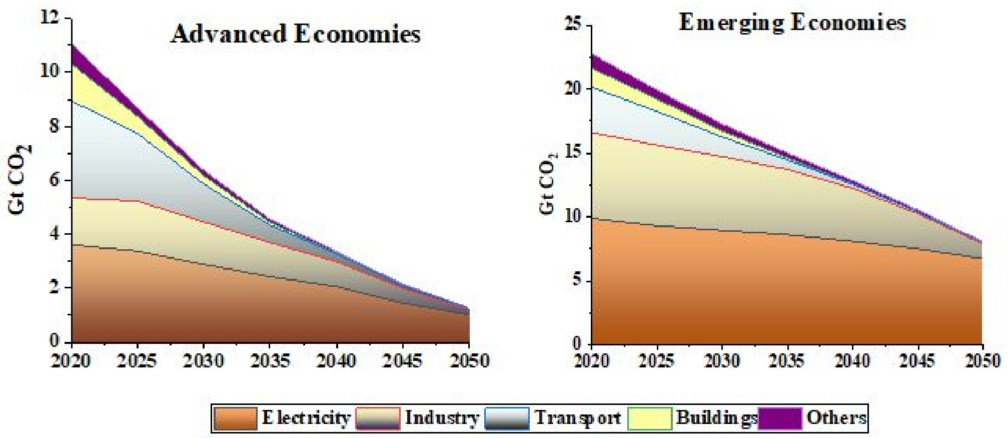


Figure S1: $CO_2$ emission trend for advanced and emerging economies until 2050 [1].

Similarly, the projected share of various energy resources like renewables, nuclear, natural gas, oil and coal towards electrical power production in advanced and emerging economies from 2020 to 2050 is illustrated in Figure S2.

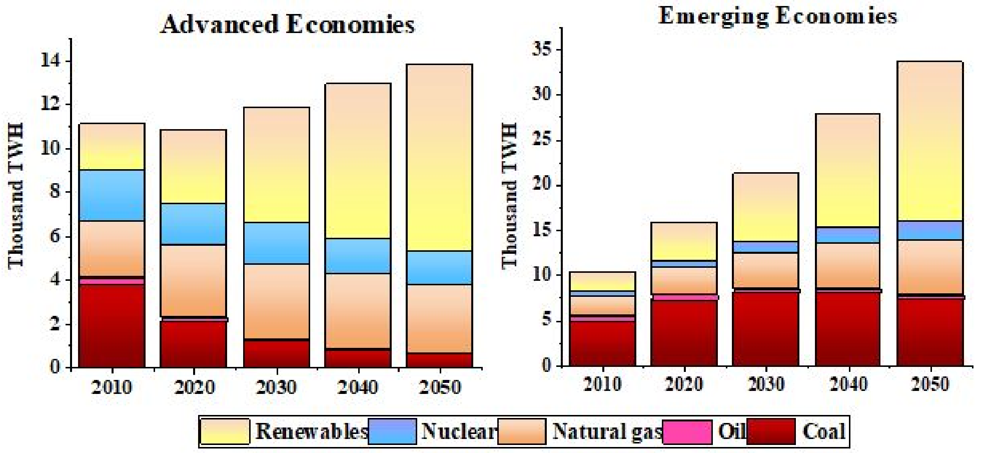

Figure S2. Electricity generation trend by renewables, nuclear, natural gas, oil, and coal till 2050 [1].

**Schematic Diagram of power plant operation**

The operation of coal power plant can be briefly explained by the working of steam cycle and flue gas system as shown in Figure S3. Coal coming from the coal hopper & feeder is pulverized in the coal mills and carried to the furnace by primary air that is supplied by the primary air fan (PAF). Secondary air is provided by forced draft fan (FDF) that is preheated in the air-preheater (APH) and is supplied to the furnace to support the combustion and controlling the $NO_x$. The fuel combustion in the furnace produces high temperature flue gas that exchanges heat with the heating surfaces like water walls, superheater, reheater, economizer etc. installed in the boiler. Upon leaving the boiler, flue gas is passed through low-temperature economizer to recover the heat and then goes to electro-static precipitator where fly ash is removed. Later, flue gas is pressurized by induced draft fan (IDF) and is passed through flue gas desulphurization (FGD) scrubber to remove $SO_2$ from it and cleaned gas is discharged to the ambient environment.

The steam cycle starts from the condensate collected in the condenser that is pumped by condensate pump and is passed through low pressure (LP) heater that works on the steam extractions from LP turbines. From LP heater, condensate goes to deaerator to remove its entrapped gases and then, is pressurized by feed water pump. The feed water then passes through high pressure (HP) heater to be preheated before entering the economizer. From economizer, feedwater goes to water walls installed in the furnace and then, moves to superheater to increase the thermal conditions of the steam. The high pressure and high temperature steam as regulated by governing valve enters the HP turbine and expands in the multi-stages of the turbine. The exhaust of the HP turbine is sent to the reheater to recover the steam temperature and then, the reheated steam is made to expand in intermediate pressure (IP) turbine. The exhaust of IP turbine is directed towards the LP turbines A & B for further expansion and finally, the steam is condensed in the condenser and the cycle continues. The expansion of steam in the steam turbines rotates the turbine shaft that is coupled with the generator and thus, the electricity is produced in the generator. The location of the sensors / input variables selected to model the HP turbine efficiency are represented by numbers on Figure S3.

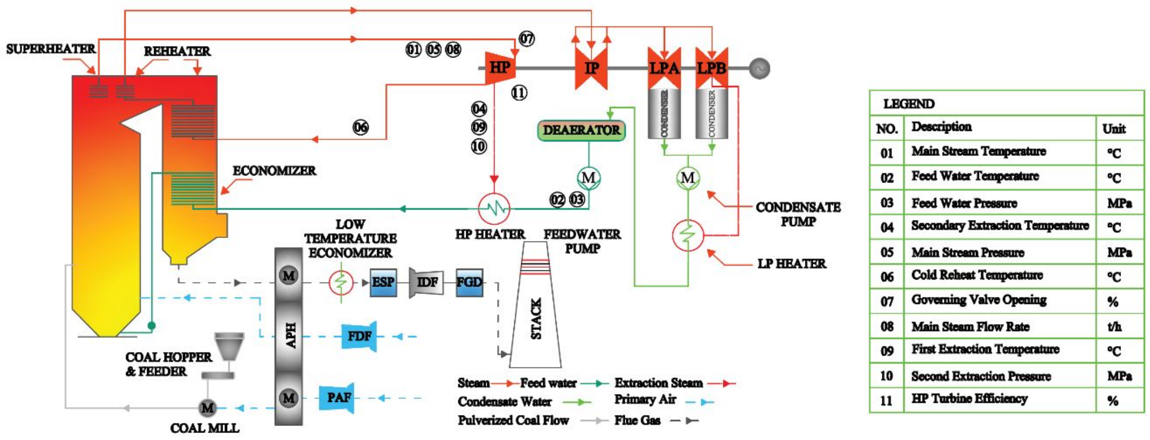


Figure S3. Schematic diagram of the working of coal power plant.

**Architecture of artificial neural network**

A typical structure of artificial neural network (ANN) consists of three layers; input layer, hidden layer and output layer as shown in Figure S4(a). The information received at the input layer is forwarded to hidden layer where it is processed and transmitted to the output layer. The subsequent information processing at the output layer computes a value which is compared with the true value to be simulated, and an error is calculated. The backpropagation of error tunes the training parameters of the ANN network and keeps iterating the process until the stopping criteria is met and an ANN network is developed.

A data split of 0.8, 0.1 and 0.1 is used for assigning the data to training, testing and validation dataset respectively. The testing and validation datasets are used to evaluate the predictive performance of the ANN during the training and the parameters (weights and biases) are updated to improve the functional mapping among the variables. Referring to Figure S4(b), mean-squared-error (MSE) is reduced significantly in the beginning for the training, testing and validation dataset. Later, as the epochs progress, the marginal decrease in the MSE is observed and the network stopped training when the maximum failure count for the validation case (20) are achieved. This is established to avoid overfitting on the data and to ensure that the ANN model may have generalized learning to predict the input conditions. Upon the network training, loss value calculated for the three training, testing and validation datasets which are as follows: 0.16, 0.15 and 0.16 respectively. The loss value for the developed ANN model is reasonably small indicating good error convergence achieved to model the HP turbine efficiency on the input variables.

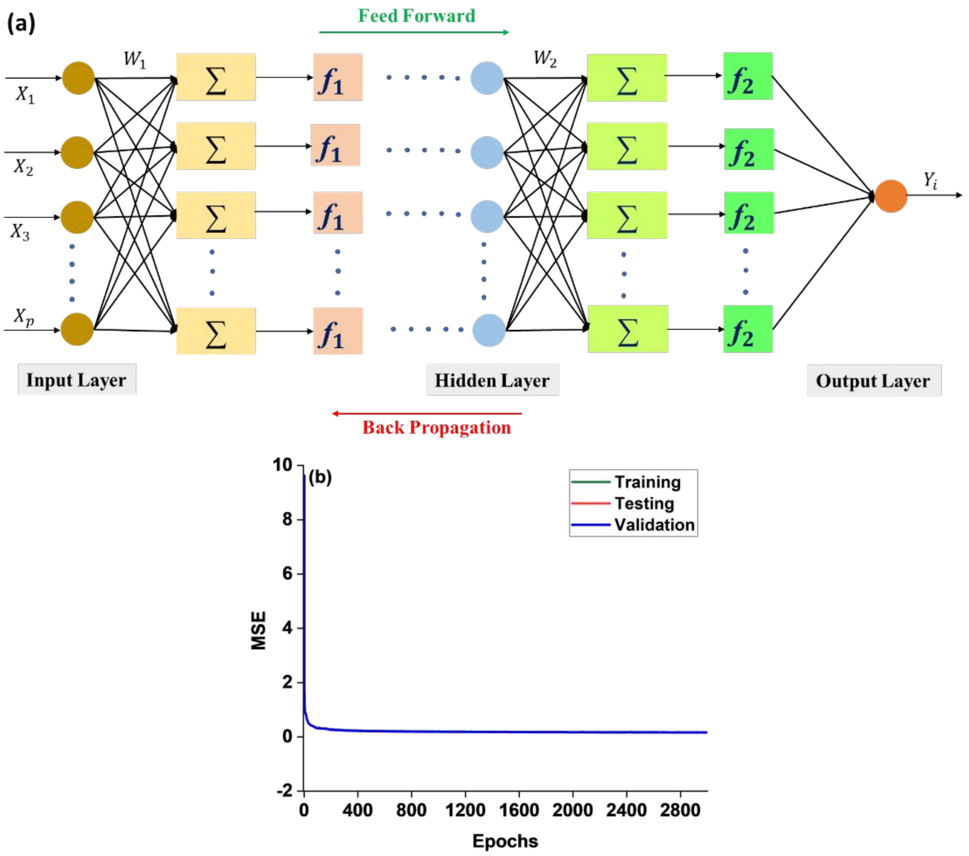


Figure S4. (a) Architecture of ANN, and (b) error convergence of the ANN for training, testing and validation dataset.

## Hyperplane geometry in support vector machine

The data separation by a linear hyperplane constructed by SVM is shown in Figure S5(a). The closest points which lie on the boundaries are called the support vectors, whereas margin is defined as the distance between the boundaries. During training, SVM attempts to develop a hyperplane so that margin is maximized, known as maximum margin width. Figure S5(b) shows the hyperplane's circular geometry for the data, which is not linearly separable. As the complexity and dimensions of the data in the input space increase, it becomes difficult to fit the hyperplane across the data. Therefore, data is projected into higher dimensional space with the help of the Kernel function [2]. Figure S5(c) illustrates that the training data in higher dimensional space becomes linearly separable even though it was non-linearly distributed in lower-dimensional feature space.

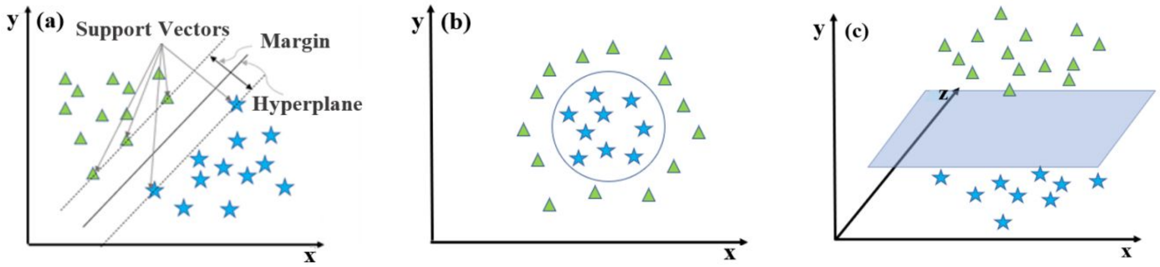


Figure S5. Data segregation topology in SVM for (a) linear, (b) non-linear 2-dimensional, and (c) higher-order data projection

## Hyperparameters tuning for SVM network

The minimum MSE (error computed between the predicted values of model and the actual responses) variation for the selected values of hyperparameters under iterative training of SVM is illustrated in Figure S6. The MSE error is decreased during the iterative training of the SVM model. It is found that minimum MSE is achieved for the optimized values of $C$ and ε as 42.7095 and 0.0052464 respectively. Similarly, four more SVM models were trained, and the better trained SVM model was selected on the bases of external validation test.

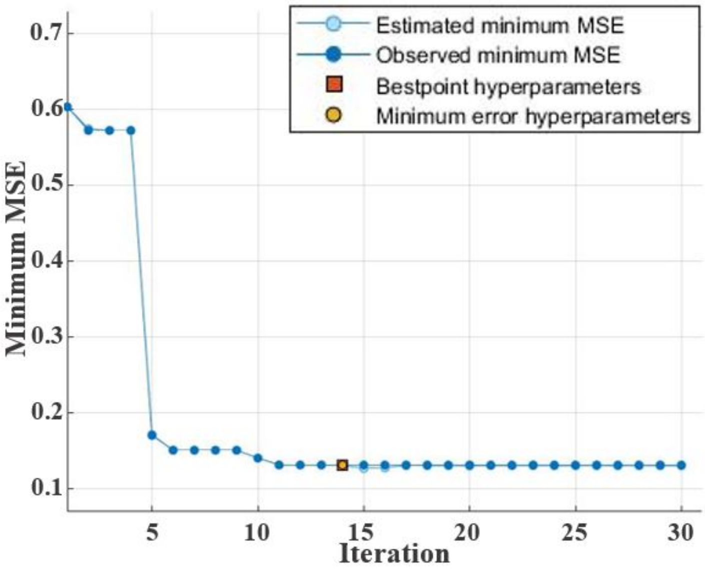


Figure S6. Iterative training of SVM model under hyperparameters tuning.

**Reference**


1. IEA, *Net Zero by 2050. 2021, IEA: Paris. p. 224.*
2. Vanajakshi, L. and L.R. Rilett. *A comparison of the performance of artificial neural networks and support vector machines for the prediction of traffic speed*. in *IEEE Intelligent Vehicles Symposium, 2004*. 2004. IEEE.
